# Open randomised prospective comparative multi-centre intervention study of patients with cystic fibrosis and early diagnosed diabetes mellitus

**DOI:** 10.1186/1471-2431-14-70

**Published:** 2014-03-11

**Authors:** Manfred Ballmann, Dominique Hubert, Barouk M Assael, Kai Kronfeld, Marguerite Honer, Reinhard W Holl

**Affiliations:** 1 Department Paediatric Pulmonology, Paediatric clinic at St Josef Hospital, Ruhr University Bochum, Alexandrinen Strasse 5, Bochum 44197, Germany; 2 Service de Pneumologie, APHP Hôpital Cochin, Paris, France; 3 Ospedale Civile Maggiore, Verona, Italy; 4 Interdisciplinary Centre for Clinical Trials (IZKS), University Medical Centre, Mainz, Germany; 5 Mukoviszidose Institut gGmbH, Bonn, Germany; 6 University of Ulm, Institute of Epidemiology and Medical Biometry, Ulm, Germany

**Keywords:** Cystic fibrosis, Diabetes mellitus, Lung diseases, Genetic diseases, Inborn, Repaglinide, Insulin, HbA1c, Clinical trial

## Abstract

**Background:**

Diabetes mellitus may be present in patients with cystic fibrosis starting in the second decade of life. The prevalence increases rapidly with increasing age. As life-expectancy increases in cystic fibrosis, cystic fibrosis related diabetes will be diagnosed more frequently in the future.

Up to date, no data are available to answer the question if cystic fibrosis related diabetes should always initially be treated by insulin therapy. Missing data regarding oral antidiabetic treatment of newly diagnosed cystic fibrosis related diabetes are an important reason to recommend insulin treatment. Several centres report the successful management of cystic fibrosis related diabetes using oral anti-diabetic drugs at least for some years. Oral therapies would be less invasive for a patient group which is highly traumatized by a very demanding therapy. Based on an initiative of the German Mukoviszidosis-Foundation, the present study tries to answer the question, whether oral therapy with repaglinide is as effective as insulin therapy in cystic fibrosis patients with early diagnosed diabetes mellitus.

**Methods/Design:**

In all cystic fibrosis patients with an age of 10 years or older, an oral glucose tolerance test is recommended. The result of this test is classified according to the WHO cut off values. It is required to have two diabetes positive oral glucose tolerance tests for the diagnosis of diabetes mellitus.

This study is a multi-national, multicentre, open labelled, randomized and prospective controlled parallel group’s trial, with 24 months treatment.

The primary objective of this trial is to compare the glycaemic control of oral therapy with Repaglinide with insulin injections in patients with cystic fibrosis related diabetes after 2 years of treatment.

The trial should include 74 subjects showing cystic fibrosis related diabetes newly diagnosed by oral glucose tolerance test during annual screening for cystic fibrosis related diabetes.

Patients are randomised by central fax randomisation.

Primary endpoint is mean HbA1c after 24 months of treatment. Secondary endpoints are change in FEV1% predicted and change in BMI-Z-score.

**Discussion:**

There is only one prospective study comparing oral antidiabetic drugs to insulin in the treatment of CFRD without fasting hyperglycaemia. The results regarding BMI after 6 months and 12 months showed an improvement for the insulin treated patients and were inconsistent for those treated with repaglinide. HbA1c and lung function (FEV1%pred) were unchanged for either group. The authors compared the changes -12 months to baseline and baseline to +12 months separately for each group. Therefore a direct comparison of the effect of repaglinide versus insulin on BMI, HbA1c and FEV1%pred was not presented. According to our protocol, we will directly compare treatment effects (HbA1c, BMI, FEV1%pred) in between both groups. The actual Cochrane report regarding “Insulin and oral agents for managing CFRD” stated that further studies are needed to establish whether there is clear benefit for hypoglycemic agents. We expect that the results of our study will help to address this clinical need.

**Trial registration:**

ClinicalTrials.gov Identifier: NCT00662714

## Background

Diabetes mellitus may be present in patients with cystic fibrosis (CF) starting in the second decade of life. The prevalence increases rapidly with increasing age reaching around 30-40% in the fourth decade. As life-expectancy increases in CF, CF-related diabetes (CFRD) will be diagnosed more frequently in the future [1].

Negative consequences of CFRD include: catabolic metabolism, weight loss, more frequent/more severe infections, deterioration of pulmonary function, reduced life-expectancy and diabetic micro vascular complications [2].

Up to date, insulin is the only recommended treatment of CFRD, whether newly diagnosed or not. But no data are available to answer the question if CFRD must always initially be treated by insulin [3]. Several centres report the successful management of CF-related diabetes using oral anti-diabetic drugs at least for some years [4,5]. Oral therapies would be less invasive for a patient group which is highly traumatized by a very demanding pharmaceutical therapy (including antibiotics, pancreatic enzymes, bronchodilators, mucolytic agents) in addition to physiotherapy. Based on an initiative of the German CF-Foundation (Mukoviszidose e.V.), the present study tries to answer the question, whether oral therapy with repaglinide is as effective as insulin therapy in CF patients with early diagnosed diabetes mellitus.

### Primary objective

The primary objective of this trial is to compare the glycaemic control on oral therapy with repaglinide tid with that on insulin therapy with three daily injections of regular Insulin over 2 years in patients with CF and CFRD.

### Secondary objectives

The secondary objectives of this trial are to compare the two treatment regimen (oral therapy with Repaglinide and insulin therapy with regular insulin) in terms of efficacy and safety.

The efficacy objectives are change in glycaemic control after 12 months of treatment, glycaemic control assessed by glucose profile done monthly (6 values), change in nutritional status and pulmonary function at 12 and 24 months, as well as the need for antibiotic treatments.

The safety objectives are the incidence and quality (mild, severe, other) of hypoglycaemic episodes and adverse events.

## Methods/Design

### Sponsor

The Mukoviszidose Institut gGmbH is the sponsor for this trial. Collaborators are Novo Nordisk, Mucoviscidose-ABCF2, Vaincre la Mucoviscidose and Assistance Publique - Hôpitaux de Paris. The protocol and all trial documents have been approved by the ethic committees responsible for the respective trial sites. The first vote was received from the ethical committee of the MHH Hannover (22.08.2001, No. 2739). The responsible competent authorities gave approval (so far applicable) under EudraCT Number 2006-001254-27 [for France and Italy], BfArM Number 4019636 [for Germany] and BMSG Number 21.405/219 [for Austria]. The trial was public registered under ClinicalTrials.gov Identifier NCT00662714.

### Eligibility criteria

Informed consent was to be obtained before any trial-related activities. CF was diagnosed by positive sweat test and/or two disease-causing Cystic Fibrosis Transmembrane Conductance Regulator (CFTR) mutations. Patients had diagnosed diabetes mellitus by OGTT done less than six months before (one year before in Italy). The subjects were over 10 years of age (12 years in France and Italy). Diabetes was confirmed by a second OGTT test. Patients in France needed in addition fasting blood glucose levels ≤170 mg/dl. Patients have been excluded with diagnosis of type 1 diabetes or diabetic keto-acidosis (blood glucose > 350 mg/dl and arterial pH < 7.25). Already treated diabetes mellitus by oral antidiabetic medication or insulin led to exclusion of these patients. Severe pulmonary insufficiency with FEV1 < 25% (France and Italy) or < 40% (Germany and Austria), participation in another clinical trial less than one month before inclusion in this trial, severe liver insufficiency or PEG/gastric tube/total parenteral nutrition for more than 4 weeks were not at inclusion. Patients must not be pregnant and in France and Italy breast-feeding or the intention of becoming pregnant or not using adequate contraceptive measures were not allowed. Patients after transplantation or in France patients which have been planned for transplantation could not be included. For Germany subjects receiving concomitant medication known to interfere with repaglinide therapy were not included. In France and Italy proliferative retinopathy, type 2 diabetes, severe renal insufficiency, history of alcoholism, drug abuse, psychiatric disease or personality disorders likely to invalidate voluntary consent or to prevent good compliance with the trial protocol, legal incapacity or limited legal capacity, known or suspected allergy to the insulin or any compositional component, known or suspected allergy to repaglinide or receiving concomitant medication known to interfere with glucose metabolism prevented inclusion in this trial.

### Trial conduct

In all CF-patients 10 years or older, an oral glucose tolerance test (OGTT) following standard WHO recommendations has be performed. Oral medication was postponed until after the test, however, inhalation therapy could be performed prior to the test. During acute infections or exacerbations of a chronic infection, the test was postponed for 3 to 4 weeks until acute symptoms improved. OGTT were classified as normal glucose tolerance, impaired glucose tolerance/impaired fasting glucose or diabetes mellitus according to the WHO cut off values [6].

### Randomization

After detailed information by the physicians of the local CF centre, the patient or legal guardian decided to give written informed consent to participate in the study. The trial was open-labelled in order to avoid use of double-dummy technique which would have required an unacceptable increase in the number of injections for the patients. The patients were allocated to the treatment regimen according to a randomisation list generated by a geigy random number table by central fax randomisation. Stratification was conducted according to gender and two age groups (10–15 years, >15 years). Every randomised patient should have been observed for at least 2 years and be included in the final analysis (“intention-to-treat” analysis).

The trial procedures are summarised in the following Table 1.

**Table 1:** Trial procedures

**Visits/months**	**VS**	**V1**	**V2**	**V3**	**V4**	**V5**	**V6**	**V7**	**V8**	**V9**	**End**
		**0**	**3**	**6**	**9**	**12**	**15**	**18**	**21**	**24**	
Informed consent	+										
Selection/exclusion criteria	+										
Randomisation		+									
Inclusion/exclusion criteria		+									
Demographic data	+										
Medical history	+										
Physical examination	+	+	+	+	+	+	+	+	+	+	+
Concomitant illness and medication	+	+	+	+	+	+	+	+	+	+	+
Blood glucose profile			+	+	+	+	+	+	+	+	+
HbA_1c_		+	+	+	+	+	+	+	+	+	+
Pulmonary function		+	+	+	+	+	+	+	+	+	+
IV therapy (antibiotics)		+	+	+	+	+	+	+	+	+	+
Adverse events		+	+	+	+	+	+	+	+	+	+
Hypoglycaemic episodes			+	+	+	+	+	+	+	+	+
Drug dispensation		+	+	+	+	+	+	+	+		

### Interventions

#### Therapeutic regimen repaglinide

**Figure 1:**
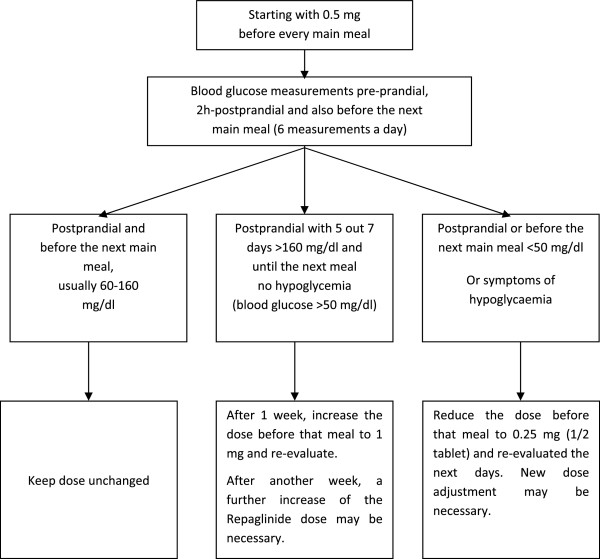
Algorithm for dose adjustment of repaglinide.

Therapy started with 3 × 0.5 mg Repaglinide before the main meals. If one meal has been skipped or consisted only of carbohydrate-free food, tablet intake was not recommended. Therapy adjustment (dose increase to 3 × 1 mg or dose reduction to 3 × 0.25 mg) occurred basing on blood glucose level measured 2 hours after a meal. The target value should have been 70–160 mg/dl. If the majority of values were above 160 mg/dl, the dose should have been further increased but not earlier than one week after start of therapy. The maximum dose in this study should have been 12 mg (3 × 4 mg). If low blood glucose levels (hypoglycaemia symptoms or blood glucose level below 50 mg/dl) occurred postprandial or before the next main meal, the repaglinide dose should have been reduced (see Figure 1). Asymptomatic blood glucose levels between 50 and 60 mg/dl could have been tolerated. For some patients, different doses before breakfast, lunch and dinner could have been reasonable.

During the adjustment phase up to 6 measurements of blood glucose levels were recommended (pre-and postprandial after the main meals respectively). After a stable adjustment was achieved, this number might be reduced for every patient individually, but one postprandial measuring of blood glucose levels had to be maintained daily. Each postprandial blood glucose level had to be checked at least once a week.

Reasons for choosing repaglinide as oral anti-diabetic.

A decreased insulin secretion appeared to be part of CF-diabetes pathogenesis repeatedly. Biguanides, sensitizers and glycosidase inhibitors are only of restricted use with CF patients due to the chemicals’ hepatic or gastrointestinal side effects. In Germany, the most extensive experience with oral anti-diabetics in CF-associated diabetes mellitus therapy has been with glibenclamide [7]. Because of its longer lasting effect the risk of hypoglycaemia, especially when meals are skipped, is higher compared to repaglinide. The latter can be adapted to meals more conveniently. For these reasons glimepiride was not chosen for this study. Nateglinide is a substance whose effects profile is similar to that of repaglinide. This substance was still under approval at the date designing this trial while therapy using repaglinide had already been documented extensively. Therefore, nateglinide was not chosen.

There are individual reports of successful use of repaglinide during CF diabetes [8]; this substance is also used in the USA in a study of adult CF patients with diabetes mellitus (Principal investigator: A. Moran, MD, Minneapolis) [9].

### Therapeutic regimen insulin

**Figure 2:**
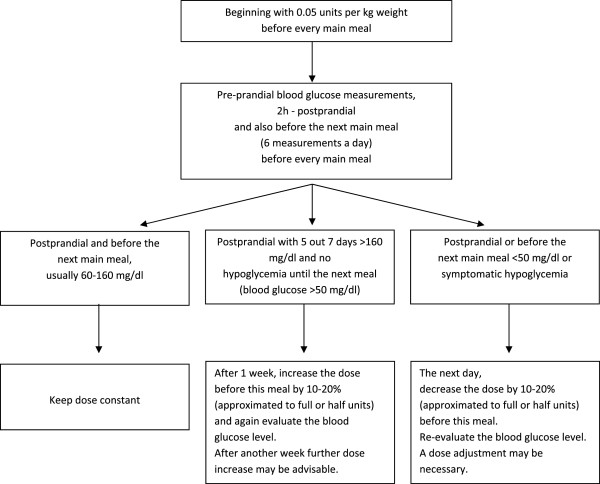
Algorithm for insulin adjustment.

Insulin therapy should have been conducted by three injections of Regular Insulin, each time immediately before the main meals. With asymptomatic patients, therapy should begin with 0.05 units per kg weight and injection. The dose was adjusted with regard to postprandial (2 hours after meal) glucose levels: The dose should be adjusted by about 10–20%, approximated to full or half units (see Figure 2). Patients injected insulin using insulin pens.

During the adjustment phase, up to 6 blood glucose measurements were recommended (each pre- and postprandial). After a stable metabolism adjustment was attained, this number could have been reduced individually; however, every postprandial level had to be checked once a week. 3 daily measurements were the minimum, especially when using higher insulin doses (>0.3 units/kg and day). Patients with varying blood glucose levels should have been educated about blood glucose corrections based on body weight and insulin demand. If the absorbable carbohydrate content of meals changed significantly on a daily basis, then a separate carbohydrate exchange unit factor for every meal based on pre- and postprandial measurements had to be determined.

### Reason for the choice of insulin therapy scheme

For insulin therapy of CF patients, conventional schemes (1–2 injections a day, fixed or varying mixtures of regular- and NPH-insulin) are advocated as well as intensified therapy schemes with 3, 4 or more injections and flexible adaptation of insulin doses to the expected food intake and the measured blood glucose level [9]. The study described here aimed at CF patients who have been diagnosed early, thus making an intensified therapy scheme unnecessary. In the case of type 2-therapy, which shows some parallels to CFRD, recent years have shown a development away from doses of premixed insulin with high NPH contents and towards pre-prandial doses of regular insulin [10]. Most CF diabetes patients show very high postprandial blood glucose levels, whereas fasting values and pre-prandial values remained normal for a long time. Therefore, a therapy of 3 regular insulin injections is advisable from a pathophysiological viewpoint. A dose of 3 injections a day for CF patients with an early diagnosis of diabetes mellitus appeared to be a viable compromise between the two aims – to apply a therapy scheme as simple as possible and to optimise a patient’s blood glucose levels at the same time.

### Diabetes education at the onset of therapy

All CF patients with newly diagnosed diabetes mellitus had to be educated intensively about food formulations, effects of food on blood glucose, blood glucose measuring, blood glucose documentation, risk of hypoglycaemia during therapy, treatment of hypoglycaemia, long-term effects of high blood glucose levels, and characteristics of diabetes mellitus with CF. Patients who have been randomised to insulin therapy had to be taught additionally about injecting, storage of insulin and dose adjustment.

### Therapy duration

Patients in both therapy strains hade to be monitored for 2 years; every 3 months, a standardised examination and documentation was intended.

### Reasons for study duration

No relevant short-term changes have been expected among early diagnosed patients, especially concerning weight development and lung function [11]. Evidence of successful diabetes therapy with an oral anti-diabetic stretching over 2 years was planned to show constitute clinically relevant results compared with a shorter duration.

### Discontinuation criteria

The therapy in one of the therapy strains should have been considered ineffective if one of the following discontinuation criteria had been met: No acceptable metabolic control obtainable over a time span of 3 months regardless of dose adjustment according to protocol and therapy compliance; occurrence of non-tolerable undesired side effects under therapy; transaminase increase to 3 times the initial value; non tolerable allergic reactions; discontinuation of study by patient/family; se of systemic steroids necessary; desired or occurring pregnancy; PEG/nasal tube feeding/completely parental alimentation of at least 4 successive weeks or transplantation.

### Therapy alternatives when reaching discontinuation criterion

a. Insufficient metabolism control during therapy with repaglinide.

Generally, a shift to insulin would have been necessary; doses of 3 injections of regular insulin before meals were recommended. If fasting blood glucose levels had been high, an additional dose of intermediate/long-lasting insulin before going to sleep was advisable. A shift from repaglinide to glibenclamide would have make sense only if allergic reactions to repaglinide occurred. The use of other oral anti-diabetics could not be recommended based on current scientific knowledge.

b. Insufficient metabolic/glycaemic control during therapy with three injections of regular insulin.

Usually a standard intensified therapy of 4–5 injections a day was recommended.

### Outcome measures

Primary endpoint:

Primary endpoint was the change in HbA1c after 24 months of treatment.

Secondary endpoints:

Secondary endpoints were glycaemic control assessed by mean HbA1C and mean blood glucose profile; change in BMI-Z-score; change in FEV1% predicted and safety assessed by antibiotic therapy, incidence of adverse events, number of hypoglycaemic episodes, all symptomatic hypoglycaemia, hypoglycaemia that necessitates the help of other persons, hypoglycaemia with unconsciousness or coma and number of blood glucose levels below 50 mg/dl in relation to all blood glucose measurements.

### Quality assurance

Clinical on-site monitoring in all trial centres was done by personal visits of a clinical monitor according to standard operating procedures of the Interdisciplinary Centre for Clinical Trials (IZKS) Mainz starting 2008. The monitor checked the informed consent forms and reviewed the entries into the case report form (CRF) on the basis of source documents. The physician allowed the monitor access to at all essential documents and provides support to the monitor. The IZKS Mainz assisted the physician to conduct the study according to the protocol as well as to regulatory and ethical requirements.

### Data management

University of Ulm conducted the data management of the trial. All protocol-required information collected during the study had been entered by the investigator, or a designated representative in the CRF. CRF data were entered by University of Ulm into the database. During the study, data were exported into the statistical analysis system and checked additionally for plausibility, consistency and completeness. Based on these checks, queries have been produced. Any missing data or inconsistencies were reported back to the respective site and clarified by the responsible investigator. After all corrections were done, the database was closed and used for statistical analysis. All collected data have been processed according to the German Data Protection Law and handled in strictest confidence.

### Power calculation/analysis

Which number of patients was necessary to show a significant difference between therapy strains of 80% power on the one hand and p <0.05 on the other? Retrospective HbA1c data from a database of the CF centre at Medical School Hannover were used. Measurements of n = 132 HbA1c within the first two years after diabetes diagnosis of n = 44 patients older than 10 years at the date of diagnosis resulted in an average value of HbA1c = 5.6 ± 1.5 (average value ± SD). Within a similar group (older than 10 years) but without diabetes n = 763 measurements yielded an HbA1c of 4.6 ± 0.68 (average value ± SD). A difference of 1 (which is in the range of individual variations during treatment especially in youngsters and young adults) between therapy groups was considered relevant [12]. Calculation with *n Query®* of necessary patients for each group was done with these parameters: Two group *t*-test of equal means (equal n’s) 1; Test significance level, α 0,050; 2 sided test; Group 1 mean, μ_1_ 5,600; Group 2 mean, μ_2_ 4,760; Difference in means, μ_1_ - μ_2_ 1,000; Common standard deviation, σ 1,500; Effect size, δ = |μ_1_ - μ_2_|/σ 0,667; Power 80%. Based on the above assumptions the number of patients in each group was calculated as n = 37. Over a treatment time of 2 years, about 30% of patients were assumed to drop out of the study. Therefore, n = 111 patients have been necessary at the beginning of the study to ensure a number of n = 74 remaining patients at the end of the study.

For analysis, the differences of primary parameter HbA1c repeated measures-ANOVA will be applied. The one-sided zero hypothesis (Repaglinide is not worse than insulin) will be dismissed if a p value under 0.05 occurs. The two therapy groups will be compared concerning secondary parameters statistically using the fisher exact test (comparison of discrete events), the Wilcoxon (non-parametrically distributed parameters) and the *t*-test (parametrically distributed parameters). For comparison of drop-outs between the treatment stains a Cox proportional-hazard-ratio model will be applied.

## Discussion

As many other treatments the use of oral antidiabetic drugs in patients with CFRD isn’t officially approved. Guidelines recommend insulin as first line treatment [2]. Nevertheless there are many centers which use oral antidiabetic drugs for treatment of CFRD [3]. The reasons for this off label use might be the hope to reduce the treatment burden which is even without CFRD very high in CF [12,13]. The only published prospective study comparing oral antidiabetic drugs to insulin in the treatment of CFRD without fasting hyperglycaemia [9] followed patients for 12 months only. The results regarding BMI after 6 months and 12 months were inconsistent for those treated with repaglinide and showed an improvement for the insulin treated patients. There was no change in HbA1c or lung function (FEV1%pred) for either group (insulin, repaglinide and placebo). An important difference to the present study protocol was the way how effects of repaglinide and insulin were evaluated. According to our protocol, we will directly compare treatment effects (HbA1c, BMI, FEV1%pred) in between both groups. In the recent published study the authors compared the changes -12 months to baseline and baseline to +12 months [9] separately for each group. Therefore a direct comparison of the effect of repaglinide versus insulin on BMI, HbA1c and FEV1%pred was not presented. The actual Cochrane report [3] regarding “Insulin and oral agents for managing cystic fibrosis-related diabetes” stated that further studies are needed to establish whether there is clear benefit for hypoglycemic agents. We expect that the results of our study will help to address this clinical need.

### Trial status

1093 CF patients with no previous diagnosis of CFRD and age of 10 years or older were screened for eligibility by at least two valid OGTTs each. For 145 patients CFRD could be diagnosed [14]. The first patient was included on 13.03.2002 and the last patient on 01.12.2009. The analysis of trial data is ongoing.

## Abbreviations

ANOVA: Analysis of variance; BfArM: Bundesinstitut für Arzneimittel und Medizinprodukte; BMI: Body mass index; BMSG: Bundesministerium für soziale Sicherheit und Generationen; CF: Cystic fibrosis; CFRD: Cystic fibrosis related diabetes; CFTR: Cystic fibrosis transmembrane conductance regulator; CRF: Case report form; CYP: Cytochrome P450; EudraCT: European clinical trials database; FEV1: Forced expiratory volume in 1 second; HbA1c: Glycosylated haemoglobin; IZKS: Interdisciplinary centre for clinical trials; NCT: National clinical trial number; OGTT: Oral glucose tolerance test; NPH: Neutral protamine Hagedorn; PEG: Percutaneous endoscopic gastrostomy; SD: Standard deviation; V: Visit; WHO: World Health Organisation.

## Competing interests

The study was funded by the Mukoviszidose e.V. (German CF-Foundation), Vaincre la Mucoviscidose (French CF-Foundation) and ABCF Association (France). CF Foundation (Mukoviszidose e.V.). The medication was a gift from Novo Nordisk, Germany.

## Authors’ contributions

MB and RH designed the trial. All authors made a substantial contribution to the trial protocol. All authors read, commentated and approved the final manuscript.

## Pre-publication history

The pre-publication history for this paper can be accessed here:

http://www.biomedcentral.com/1471-2431/14/70/prepub
